# Spatio-temporal variation of fish taxonomic composition in a South-East Asian flood-pulse system

**DOI:** 10.1371/journal.pone.0174582

**Published:** 2017-03-28

**Authors:** Heng Kong, Mathieu Chevalier, Pascal Laffaille, Sovan Lek

**Affiliations:** 1 EDB, Université de Toulouse, CNRS, ENFA, UPS, Toulouse, France; 2 EcoLab, Université de Toulouse, CNRS, INPT, UPS, Toulouse, France; University of Waikato, NEW ZEALAND

## Abstract

The Tonle Sap Lake (TSL) is a flood-pulse system. It is the largest natural lake in South-East Asia and constitutes one of the largest fisheries over the world, supporting the livelihood of million peoples. Nonetheless, the Mekong River Basin is changing rapidly due to accelerating water infrastructure development (hydropower, irrigation, flood control, and water supply) and climate change, bringing considerable modifications to the annual flood-pulse of the TSL. Such modifications are expected to have strong impacts on fish biodiversity and abundance. This paper aims to characterize the spatio-temporal variations of fish taxonomic composition and to highlights the underlying determinants of these variations. For this purpose, we used data collected from a community catch monitoring program conducted at six sites during 141 weeks, covering two full hydrological cycles. For each week, we estimated beta diversity as the total variance of the site-by-species community matrix and partitioned it into Local Contribution to Beta Diversity (LCBD) and Species Contribution to Beta Diversity (SCBD). We then performed multiple linear regressions to determine whether species richness, species abundances and water level explained the temporal variation in the contribution of site and species to beta diversity. Our results indicate strong temporal variation of beta diversity due to differential contributions of sites and species to the spatial variation of fish taxonomic composition. We further found that the direction, the shape and the relative effect of species richness, abundances and water level on temporal variation in LCBD and SCBD values greatly varied among sites, thus suggesting spatial variation in the processes leading to temporal variation in community composition. Overall, our results suggest that fish taxonomic composition is not homogeneously distributed over space and time and is likely to be impacted in the future if the flood-pulse dynamic of the system is altered by human activities.

## Introduction

Tropical freshwater systems, especially floodplain lakes and rivers, support productive fisheries, providing food and incomes for millions of people worldwide, particularly in the poorest countries [1,2]. In 1990, it was estimated that over 120 million people were involved in fisheries related activities, including capture, processing and sale of fish with 95% of them located in developing countries [3]. In Malawi, fishing activities from Lake Chilwa support about US$18 million annually while Lake Naivasha support an export-oriented agriculture valued at US$ 613–640 million [4]. Likewise, annual fish production from the Tonle Sap Great Lake (TSL) was estimated at 180,000 to 250,000 tons, representing approximately 60% of the total fish production of Cambodia [5]. This fish resource provides food for 14 million people and represents approximately 16% of the Cambodia's gross domestic product [6,7].

The TSL is the largest natural lake in South-east Asia, the largest wetland in the Mekong region, the most productive inland fisheries in the world and is a hotspot for biodiversity (i.e. it was designated as a UNESCO Biosphere Reserve in 1997), providing essential habitats for many endangered fishes and birds [8,9]. The TSL is a typical seasonal flood-pulse system and is a key element for the annual Mekong’s flood. From June until September, the lake fills and the water level increases from 1–2 meters up to 10–15 meters [10]. In September, the TSL reverses its flow direction toward the Tonle Sap River (TSR) until the end of February causing the water level to drop to its minimum level in April and May.

Seasonal flood-pulse dynamic influences many ecological and environmental processes by causing lateral connectivity to adjacent floodplain habitats and by influencing water quality and nutrient dynamics, thus influencing the life-cycle of many organisms [11]. Indeed, lateral connectivity is a key element for many fish and other aquatic species because it provides resources and spawning habitats favoring productivity and biodiversity, which may in turn affect ecosystem stability and resilience to perturbations [12]. Consequently, large spatio-temporal variations in community compositions are expected within flood-pulse systems. Understanding what are the factors involved in these variations may help adapt conservation strategies to promote biodiversity and maintain their value as a livelihood.

Studies focusing on the determinants of spatio-temporal variations of fish communities have mostly been conducted on temperate systems (e.g. [2,12–14]). The paucity of studies conducted on tropical systems represent a large gap regarding our understanding of their functionning because tropical systems differ in various ways from temperate ones. For instance, tropical lakes are usually subject to indiscriminate fisheries (e.g. all species and size classes are targeted) whereas fisheries in temperate lakes are strongly reglemented. Consequently, the patterns highlighted in temperate lakes may not hold in tropical ones. Studying community composition, how they vary spatially and temporally and what are the determinants of these variations is therefore an important step toward a better understanding of the functionning of tropical ecosystems [15]. This is of utmost importance if we are to better manage these ecosystems which sustain important biodiversity and fisheries.

Indeed, studying the variation in species composition among sites (i.e. spatial beta diversity) and seasons (i.e. temporal beta diversity) may help improve our understanding of the processes that generate and maintain biodiversity [16]. For instance, according to the niche theory, sites with similar environmental conditions should harbor similar species whereas the opposite is expected for sites with different environmental conditions [17]. Thus, if environmental conditions are similar within a given area low beta diversity is expected whereas the opposite is expected if environmental conditions are spatially hetrerogeneous. In this study, our aim was (1) to characterize the temporal variation in the spatial composition of fish communities among six sites within the TSL during 141 weeks, spanning two complete hydrological cycles and (2) to identify the determinants of the temporal variations in the contribution of site and species to spatial variation in community composition. For this purpose, we used data collected at six locations from a community catch monitoring program conducted from 2012 to 2014. For each week, we quantified the spatial variation of fish community composition (beta diversity) and partitioned it into local contribution (LCBD) and species contribution (SCBD). We then used linear models to explore how temporal variations of LCBD and SCBD values varied depending on the water level, the species richness and the species abundance.

## Material and methods

### Study area

**Fig 1:**
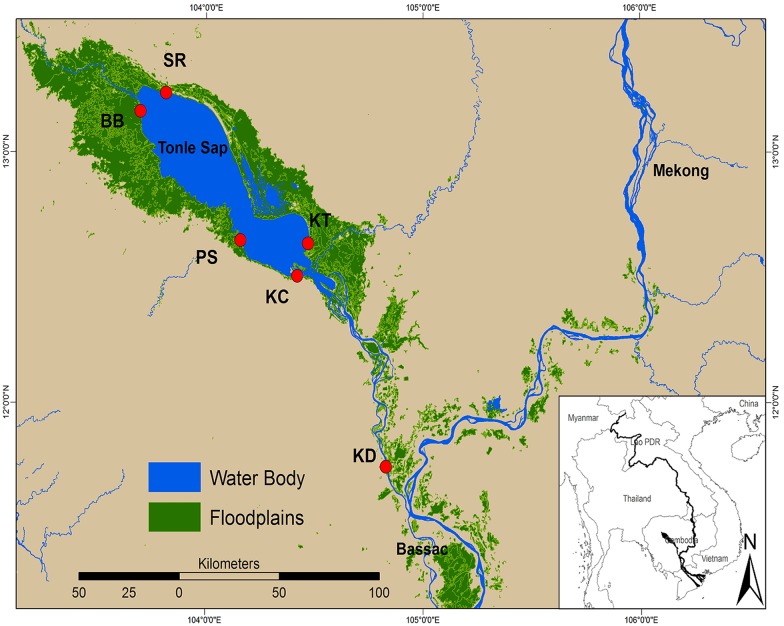
Localization of the six sampling sites. SR = Siem Reap; BB = Battambang BB; KC = Kampong Chhnang; KT = Kampong Thom; PS = Pursat; KD = Kandal. KD is located within the Tonle Sap River (TSR) whereas the five other sites are located within the Tonle Sap Lake (TSL).

The TSL is located in the central part of Cambodia (Fig 1) and is the largest natural freshwater lake of South-east Asia. It covers an area of approximately 0.25 million hectares during the dry season and an area estimated between 1.0 to 1.3 million hectares during the peak flood in the wet season. The TSL is connected to the Mekong in its southern part by a 120 km long river, the Tonle Sap River (TSR), which serves as an inlet and outlet for water fluxes. The cycle of the water level of the TSL can be divided into four phases. The first phase, the rising season, lasts from July to early September and is characterized by a strong water feed coming from the upper Mekong through the TSR. During this phase, the water level of the lake increases by about 70%. The second phase, the flooding season, occurs from the end of September to early October, and corresponds to a phase where about 1.25 million hectares of forest and agricultural land are submerged. At this time, the water level may attain up to 15 meters. The third phase, the receding season, occurs from the end of October to February and corresponds to the reversal of the river flow from the TSL through the TSR, thus leading to a decrease of the water level of the lake. Finally, the fourth phase, the dry season, lasts from April to May, and corresponds to a period where the water level is the lowest (one to two meters).

### Data collection

The fish data used in this study were derived from the Mekong River Commission (MRC), under the Assessment of Mekong Fisheries Component of the MRC Fisheries Program. The catch monitoring methods were derived from the MRC’s regional monitoring program on fish abundance and diversity in the Lower Mekong Basin. Fish catches were monitored at five sites located around the TSL (Fig 1). Two sites (Siem Reap [SR] and Battambang [BB]) were located in the northern part of the lake while three sites (Kampong Chhnang [KC], Kampong Thom [KT] and Pursat [PS]) were located in its southern part. Fish catches were monitored at another one site located in the TSR (Kandal [KD]). Each site was monitored following a community catch monitoring program conducted on a daily basis from January 2012 to May 2014, thus covering two complete hydrological cycles. The catch monitoring methods were derived from the MRC’s regional monitoring program on fish abundance and diversity in the Lower Mekong Basin [18]. The catches were performed by 18 local fishermen (3 fishermen per sites) using gillnets with 2 to 6.5 cm mesh sizes to capture as many species as possible. Fishes were identified to species level and counted. For unidentified individuals, the identification was performed later by a professional taxonomist in the laboratory. All fish records were collected monthly from fishermen and cross-checked by research officers to confirm the identifications following [19]. For further information on fish collection see [20]. Water level was measured in two locations; within the lake (PS) and within the river (KD).

The data were collected out in strict accordance with the Cambodian Fisheries Law on small-scale fishing. None of the studied species are classified as either endangered or protected according to the IUCN red list.

### Data analysis

Daily data were aggregated into weekly data to reduce the influence of rare or occasional species on the analyses, thus resulting in 141 weekly catch data for each site.

#### Spatio-temporal variation of fish communities: contribution of sites and species

For each week, we computed the total variance of the site-by-species community matrix as an estimate of beta diversity (BD_Total_) and then partitioned this measure into Local Contribution to Beta Diversity (LCBD) and Species Contribution to Beta Diversity (SCBD), following [16]. LCBD are comparative indicators of the ecological uniqueness of the sampling units and indicate how much each site contributes to beta diversity. Thus, a site with average and common species composition is expected to have a value of zero whereas large values indicate sites with different communities. Such large values may either indicate a site with a high conservation value or, on the contrary, degraded or species poor sites with a need for restoration. On the other hand, SCBD indices indicates how much each species is contributing to beta diversity. Thus, a species present in all assemblages has a value of zero whereas species with large values are those that are present in only a few locations. LCBD and SCBD values were computed for each week from community composition matrices transformed using the Hellinger transformation (i.e. a measure of the dissimilarity in the species composition among locations).

To test for differences in LCBD values computed for each week between the six sites, we used Kruskall-Wallis non-parametric analysis of variance followed by multiple comparisons Tukey post-tests to test for differences between each pair of sites. We further used a hierarchical cluster analysis to determine whether LCBD values calculated for each week and the different sites (i.e. the sampling units) could be grouped based on their similarity. We used the Euclidian distance as a measure of similarity among the sampling units and sampling units were then aggregated using the Ward's method. Finally, compositional changes in fish communities were examined using non-metric multidimensional scaling which is a rank based method attempting to represent the pairwise dissimilarity between sampling units in a two dimensional space.

#### Determinants of temporal variation in LCBD and SCBD values

We used multiple linear regressions to explain temporal variation in LCBD values at each site. Three variables were included as predictors in each model (one for each site): the site specific richness, the local abundance (i.e. the sum of abundances of all species) and the water level (measured at PS for sites located within the lake and measured at KD otherwise). These three variables were log(x+1) transformed prior to analysis to reduce the skewness of their distribution. A quadratic term was also included for each predictor to allow for non-linear responses. From the complete model, we used a stepwise procedure based on the Akaike information criterion (AIC) to select the predictors that best explained temporal variation in LCBD values. The model retained was the one with the lowest AIC. From the selected models, we performed hierarchical partitioning to assess the relative contribution of each predictor.

Because SCBD is based on species and not sites, we have as many time series of SCBD indices as the total number of species sampled (i.e. 242). To avoid building and interpreting 242 linear models and because of the presence of a large number of zeroes for most species (i.e. rare species), we calculated for each week the number of species that contributed to total beta diversity above the mean of the entire pool of species. This was done by centering SCBD values for each week and keeping only the species with positive signs [16]. We then used the same procedure as above and considered as predictors the water level, the overall species richness and the total abundances (i.e. measured over the six sites for each week) as well as their quadratic terms. We then used hierarchical partitioning to assess the relative contribution of each predictor.

All annalyses were performed within the R environment software [21], using the packages vegan [22], hier.part [23] and the function beta.div described in [16].

## Results

**Fig 2:**
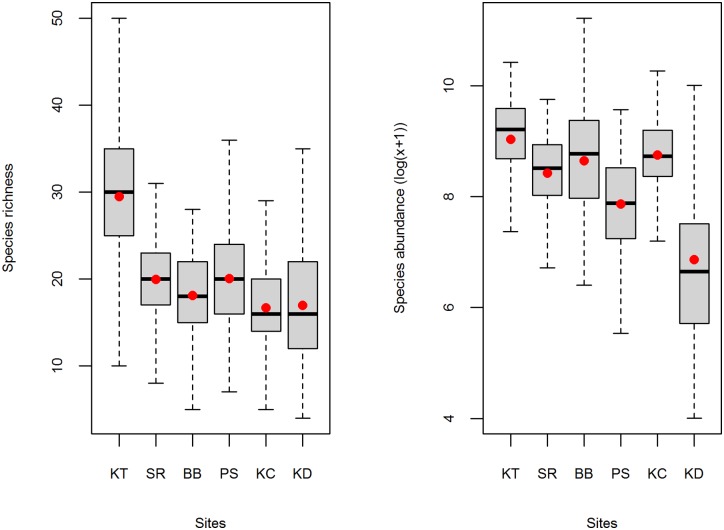
Among site variation in species richness (a) and total abundances (b). The horizontal black line represents the median whereas red points indicate the mean. For site code, see Fig 1.

Among the six studied sites and the 141-week samples, 12,455,409 individuals, belonging to 242 species, 123 genera and 49 families were captured (S1 Table). The number of species captured ranged from 2 to 53 while the number of individuals ranged from 9 to 352,594. Species richness and total abundances were both higher in KT relative to the other sites whereas there was a trend toward lower values in KD (Fig 2). In Table 1 we show the means and standard errors of the number of individuals captured within the six sampling sites for the 20 most abundant species.

**Table 1:** Means and standard errors of the number of individuals captured for the 20 most abundant species found in our samples.

Species	BB	KC	KD	KT	PS	SR
*Anabas testudineus*	28±1.8	9.6±1.3	0.1±0	58±3.2	34.1±2	34.7±3.2
*Cyclocheilichthys armatus*	6±1.1	32.4±9.1	0.1±0	56.4±4.4	1.4±0.3	51.8±2.2
*Henicorhynchus lobatus*	95.6±10.7	171.6±15.5	497.5±77.7	285±15.2	56.3±3	58.2±3.2
*Henicorhynchus siamensis*	132.2±16.1	131.8±13.7	87.8±13.9	415.3±21.5	56.6±2.7	43.5±1.9
*Labeo chrysophekadion*	2±0.2	13.8±2.7	1.2±0.1	52.5±5	41.6±1.9	2±0.3
*Labiobarbus lineatus*	13.1±1.6	142.3±15.9	-	144.4±13.9	50.2±2.3	37.3±2.3
*Labiobarbus siamensis*	34.6±6.2	9.4±2	112.3±20.7	36.3±3	44.3±2.2	41.7±2.1
*Mystus albolineatus*	55±6.3	5.7±1	-	65.7±4.1	-	1.4±0.6
*Mystus mysticetus*	73.4±6.6	35.1±3.7	0.8±0.2	38.7±3.4	44.8±2.6	74.2±3.3
*Mystus singaringan*	31.3±2.5	52.7±6.2	0.3±0	53±3.9	14.2±1.4	34.7±2.2
*Osteochilus vittatus*	95.8±7.8	67.4±5.9	0.1±0	194.2±7.2	57.2±2.4	84.1±3
*Pangasius macronema*	2±1.7	2.6±0.5	59.1±3.6	12.2±1.8	2.4±0.3	0.4±0.2
*Paralaubuca riveroi*	-	-	171.9±33.6	-	-	-
*Paralaubuca typus*	26.4±4.4	102.8±8	139.8±30	19.9±2.3	16.3±1.3	28.6±2.3
*Poropuntius deauratus*	-	130.9±9.4	-	2.6±0.8	0.9±0.5	-
*Puntioplites proctozysron*	24.3±1.6	37.9±2.4	-	88.8±5.8	44.8±1.8	126.6±4.1
*Rasbora tornieri*	11.1±2.6	219±12.5	1±0.2	1.5±0.5	1.4±0.3	7.7±1.6
*Trichopodus microlepis*	334.4±17.1	9.8±1.1	-	15.6±3	43.7±2.6	57.6±3.8
*Trichopodus trichopterus*	364.4±18.1	25.5±2.1	-	114.2±7.5	74.2±4	56.8±2.5
*Xenentodon cancila*	-	175.5±8.7	3.3±0.9	0.5±0.2	0.6±0.2	-

means and standard errors are displayed for the six sampling sites.—indicate that the species has never been detected at this site. For site code see Fig 1. For a complete list of the species see S1 Table.

### Spatio-temporal variation of beta diversity

**Fig 3:**
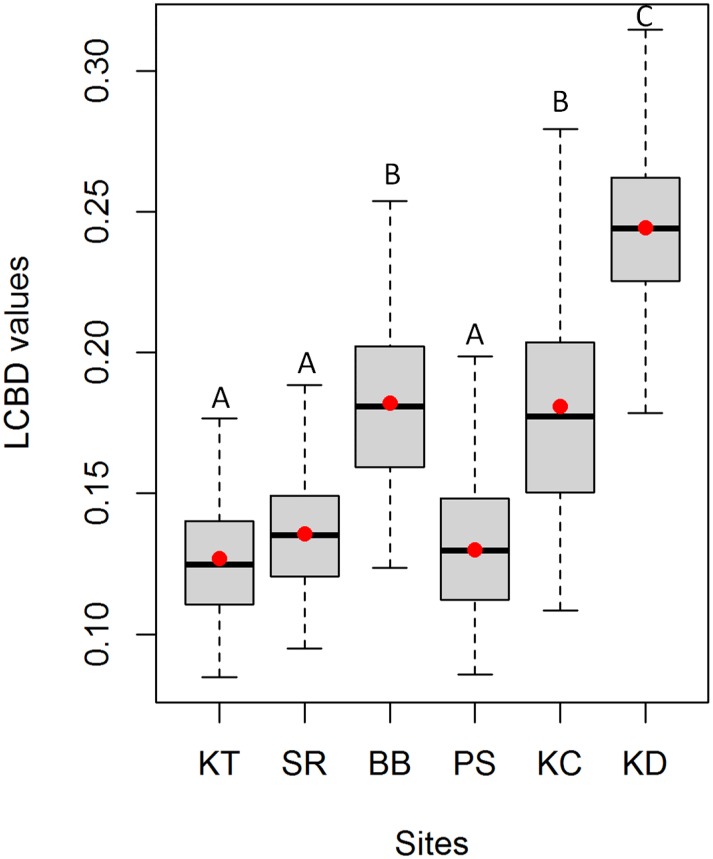
Among site variation in LCBD values. The horizontal black line represents the median whereas red points indicate the mean. The absence of common letter over of the boxplots indicate significant differences between sites in LCBD values (Tukey-post tests; p < 0.05). For site code, see Fig 1.

**Fig 4:**
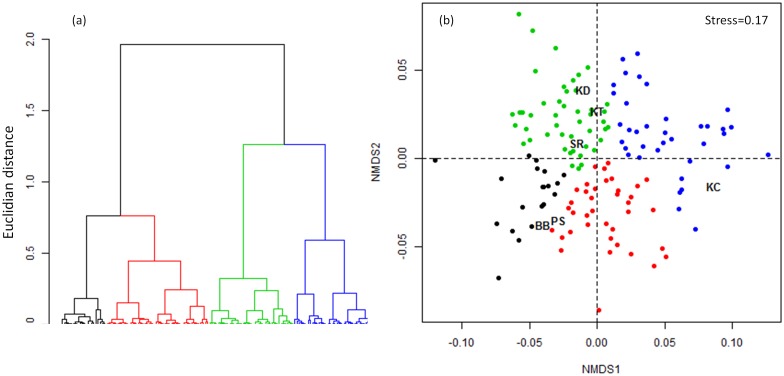
Similarity among LCBD values. (a) Hierarchical clustering of LCBD values according to their similarity (Euclidian distance) with the Ward's aggregation criteria. (b) Two-dimensional space defined by a non-metric multidimensional scaling (NMDS) approach representing the position of LCBD values and sites. In (a) and (b), the different colors represent the four groups identified by the cluster analysis. For site code, see Fig 1.

All sites and weeks confounded, LCBD values ranged between 0.08 and 0.31. Kruskall-Wallis revealed significant differences in LCBD values among sites (chi-squared = 5487.62, df = 5, P<0.001). Tukey post-tests revealed that LCBD values were higher in KD (median = 0.244; sd = 0.027) compared to the other sites (Fig 3). The lowest values were observed in KT (median = 0.124; sd = 0.021), SR (median = 0.13; sd = 0.021) and PS (median = 0.13; sd = 0.027). BB (median = 0.181; sd = 0.031) and KC (median = 0.177; sd = 0.04) displayed intermediate values (Fig 3). Based on the similarity of LCBD values, samples were grouped into four clusters (Fig 4a). The two first clusters were mainly represented by BB and PS whereas the third one mainly represented KD, KT and SR while the fourth cluster was representative of KC (Fig 4b).

**Fig 5:**
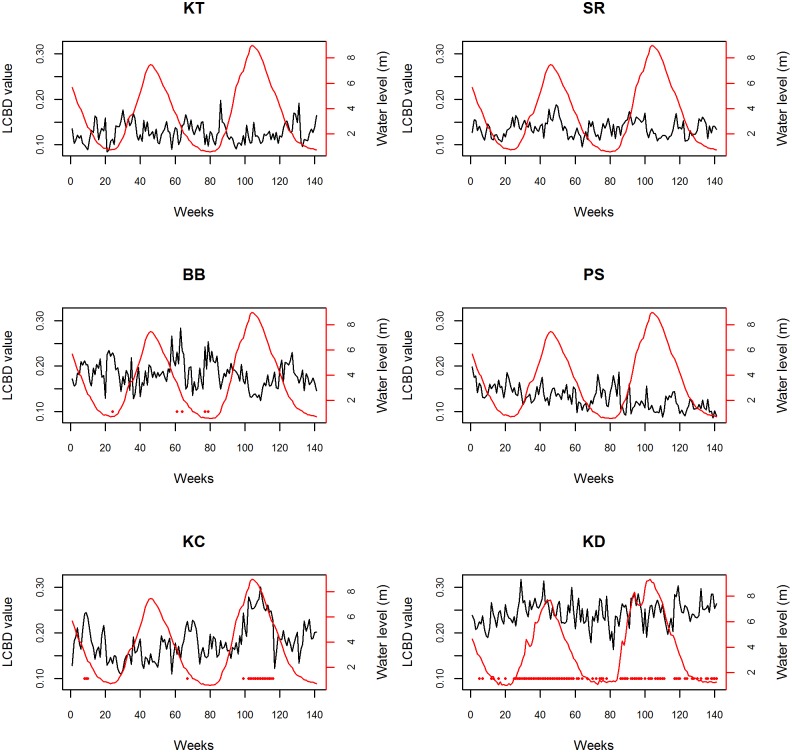
Temporal evolution of LCBD values and water level (m) over the study period for the six sites. The red dots indicate weeks with significant LCBD values (corrected for multiple comparisons). For site code, see Fig 1.

Among the 141 weeks considered, KT, PS and SR never displayed significant LCBD values, thus indicating that fish taxonomic composition within these sites do not explain spatial variation of fish community composition across the two hydrological cycles (Fig 5). In contrast, BB, KC and KD had respectively 4.2%, 14.9% and 65.2% of their weeks that displayed significant LCBD values indicating strong temporal variation regarding the uniqueness of fish community composition within these three sites.

**Fig 6:**
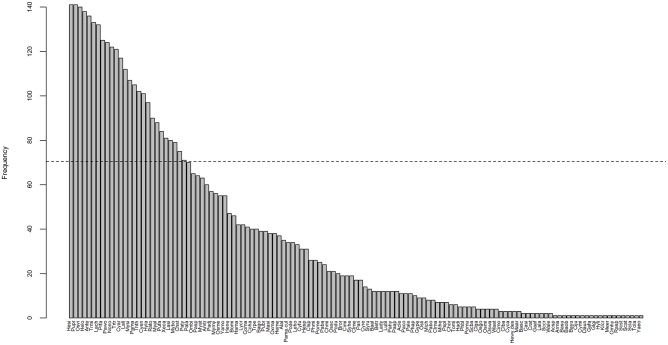
Number of weeks where species contributed to beta diversity above the mean relative to the entire pool of species. The horizontal dashed line represents 50% of weeks. For species code, see S1 Table.

More than 50% (i.e. 127) of the species contributed to beta diversity above the mean relative to the 242 species for at least one week (Fig 6). Among them, 26 species contributed to beta diversity above the mean for more than 50% of the weeks (S2 Table), thus indicating a rather stable contribution of these species to spatial variation of community composition over time. Those species were mostly non-migratory (61%) with specific habitat requirements such as permanent lakes or reservoirs (S2 Table).

### Determinants of variation in LCBD and SCBD values

Regarding temporal variation of LCBD values, we found that the influence of predictors greatly varied depending on the sites considered (Table 2). Species richness was positively related to LCBD values at BB and KC but the opposite was found at PS. Furthermore, a non-linear influence of species richness (i.e. significant quadratic term) was detected at KT, BB and KD. The influence of total abundances on LCBD values also varied depending on the site considered; a negative relationship was found at KT and PS whereas a positive one was found at KD. Also, four sites (KT, SR, PS and KD) presented a non-linear relationship between LCBD values and total abundances. The water level was linearly related to LCBD values at all sites but KC with negative relationships at KD and SR and positive relationships at KT, SR and KD. A non-linear relationship was detected at KT, SR, BB and KD. When linear and quadratic terms were considered in conjunction, the hierarchical partitioning (Table 3) revealed that the species richness had the highest independent contribution in KT (61.6%), KD (36.4%) and KC (100%), whereas total abundances presented the highest independent contribution in PS (67.4%) and SR (82.6%). Finally, the water level had the highest independent contribution in BB (74.4%). Whatever the predictor considered, their contribution varied greatly depending on sites. For instance, the contribution of total abundances to the total variance varied from 12.8% in KT to 82.6% in SR. When considered in combination, the two biotic variables (species richness and total abundances) explained more than 65% of the total variance except in BB (25.5%).

**Table 2:** Results obtained from the stepwise selection procedure.

Sites	Intercept	WL	WL^2^	SR	SR^2^	AB	AB^2^	R^2^
**KT**	0.26	4.2×10E-02	-1.6×10E-02	-	-3.9×10E-03	-3.1×10E-02	2.0×10E-3	0.27
**SR**	0.19	-3.0×10E-02	1.1×10E-2	-	-	-	6.5×10E-04	0.16
**BB**	-0.06	5.4×10E-02	-2.4×10E-02	1.7×10E-01	-3.3×10E-02	-	-	0.15
**PS**	0.03	5.0×10E-03	-	-4.1×10E-03	-	4.8×10E-02	-3.7×10E-03	0.45
**KD**	0.29	-5.5×10E-02	2.6×10E-02	-	7.6×10E-03	-4.0×10E-02	2.3×10E-03	0.29
**KC**	0.22	-	-		3.9×10E-03	-	-	0.06
**Nsp**	-295.1	-	-	202.0	-22.7	-19.7	0.8	0.29

the coefficients displayed within the table are those that were extracted from the best model (through AIC). WL = water level; SR = local species richness; AB = local abundances. (-) indicate that the predictor was not pertinent enough to explain temporal variation in the dependent variables. The models above the double line are related to temporal variation in LCBD values at each site whereas the model below the double line is related to the temporal evolution of the number of species presenting SCBD values above the mean relative to the other species (Nsp). For site code, see Fig 1.

^2^ denote quadratic terms.

**Table 3:** Hierarchical partitioning indicating the relative contribution (in percentage; %LI) of each predictor to the variance explained by the models presented in Table 2.

	%LI					
Sites	WL	WL^2^	SR	SR^2^	AB	AB^2^
**KT**	11.3	14.3	-	61.6	6.4	6.4
**SR**	7.8	9.6	-	-	-	82.6
**BB**	31.5	42.9	11.9	13.7	-	-
**PS**	2.9	-	29.7	-	32.7	34.7
**KD**	14.7	20.0	-	36.4	16.4	12.5
**KC**	-	-	-	100.0	-	-
**Nsp**	-	-	41.8	39.7	9.6	8.7

For site code see Fig 1. For predictor abbreviations, see Table 2.

Regarding the temporal variation of the number of species that contributed to total beta diversity above the mean of the entire pool of species (SCBD values above the mean after centering), we found no influence of the water level (Table 2). The selected model included a positive relationship with the species richness and a negative one with total abundances, thus reflecting the opposite effect of these two predictors on the number of species contributing to beta diversity. We further found a non-linear influence of both species richness and total abundances. The hierarchical partitioning revealed a very high contribution of the species richness with more than 80% of the variance explained (Table 3).

## Discussion

In this study, we aimed (1) to characterize the temporal variation in the spatial composition of fish communities (i.e. beta diversity) among six sites during 141 weeks, spanning two complete hydrological cycles and (2) to identify the determinants of the temporal variations in the contribution of site and species to spatial variation in community composition. We found that (1) some sites were more unique regarding fish community composition, (2) some species highly contributed to spatial differentiation of fish communities and (3) there is strong temporal variations regarding the contribution of site to beta diversity. The determinants involved in these temporal variations, their contribution and the shape (i.e. linear or quadratic) of the relationship greatly varied among sites, thus reflecting spatial variation in the processes structuring fish communities.

### Spatio-temporal variations of beta diversity

Fish community compositions are expected to vary within floodplain systems [12,24,25]. In accordance, we found large spatial variation in fish community composition reflected by differential contribution of sites to the dissimilarity between assemblages (i.e. to beta diversity). Similarly, [26] found strong spatial variations in the community composition of rock-restricted cichlid fishes in Lake Malawi which was related to the geographic distances between locations and local habitat variables. In contrast, no spatial variation in fish community composition was found within the Dianshan Lake (China) which might be explained either by homogeneous environmental conditions [27] or by strong dispersal abilities of individuals homogenizing communities over large spatial scales (i.e. "mass effect"; [28]). The large spatial variation in fish community composition found within the TSL may be explained by spatial variation in habitat availability and environmental conditions (environmental filtering) as well as by the migratory behavior of particular fish species. In accordance, a study conducted on 59 temperate lakes highlighted an influence of environmental variables in structuring fish communities both between and within lakes [29].

We found temporal variation in the contribution of sites to the spatial variation in community composition, thus suggesting strong temporal variations in local species assemblages' at large spatial scale. This result strengthen previous findings demonstrating temporal variation in community composition across seasons within the Dianshan Lake [27]. More specifically, we found that three sites (BB, KC and KD) contributed strongly to the spatial variation in community composition. For BB and KC, the uniqueness of fish communities was occasional whereas the one at KD was rather stable over time with more than 60% of the weeks being unique in terms of community composition. Such stability can be explained by the fact that KD is the only site that is not located within the lake but within the river (TSR) which is a transitional zone for species migrating back and forth between the lake and the Mekong River. The uniqueness of species assemblages at BB mostly occurred during the dry season which can be explained by the presence of particular species moving back and forth from floodplain habitats to open water habitats within the lake and also by the influence of the Sangker River, located at the north of the lake. In contrast the uniqueness of species assemblages at KC and KD was evident during the wet season and can be explained by the fact that these sites are strongly influenced by the TSR.

Over the 242 species, 50% showed a significant contribution to spatial variation of fish communities. However, this contribution greatly varied over time. This was reflected by the fact that only 10% of the 242 species showed a significant contribution to spatial variation in community composition for more than 50% of the weeks. Those species (e.g. *Mystus bocourti*, *Mystus albolineatus*, *Trichopodus microlepis*, *Anabas testudineus*, *Notopterus notopterus*, *Pristolepis fasciata*, *Channa striata*) were mostly non-migratory with specific habitat requirements. Such temporal stability suggests that these species probably depend upon the availability of critical habitats in both the wet and the dry seasons for growing or spawning. The low contribution of the remaining species to beta diversity can be explained by their widespread occurrence over the TSL, although seasonally.

### Determinants of temporal variation in LCBD and SCBD

We found that the local contributions to beta diversity (i.e. LCBD values) of the six sites displayed very different responses to species richness, total abundances and water level. Few studies have adressed the question of the determinants of temporal variations of LCBD values. Among them, a negative correlation between LCBD values and both species richness and total abundance has been reported in subtropical tree [30], dung beetle [31], cattle tick communities [32] and fish [16] communities. Such a negative relationship indicates that as sites become less species-rich, they also tend to become more unique which could be explained by the occurrence of a disturbance such as pollution. In contrast, a positive relationship may arise because of the introduction of novel species (e.g. migratory species) within communities. Here we found contrasted patterns, revealing that different processes are shaping local fish communities.

We further found that both the shape and the relative effect of the three predictors greatly varied between sites. Indeed, we found a higher contribution of biotic variables (i.e. species richness and abundances) in explaining variation in site uniqueness over time relative to the water level (abiotic variable). This contrast with previous findings showing that abiotic variables such as distance from the source, altitude and water discharge are key factors influencing species assemblages [16]. Such discrepancy may stem from the fact that we focused on the temporal variation in site uniqueness whereas previous studies [16] were interested in its spatial variation. However, the higher contribution of biotic variables does not indicate that the water level has no influence on fish communities. Instead, one can imagine an indirect effect of the water level on fish communities where a change in this variable influences connectivity to floodplain habitats, in turn leading to local changes in species abundances and richness, and ultimately leading to spatial differentiation. The non-linear relationships highlighted here are also particularly interesting because they indicate that the local uniqueness of species assemblages occur for intermediate values of water level, species richness and abundances. At both extreme of the gradient, communities are therefore more homogeneous which can be explained by the dominance of large scale processes [28]. For instance, water level reduction has been shown to influence community assemblage by influencing local individual abundances and by making it possible for species to colonize new local habitat patches [33], a process that can lead to community homogenization. Likewise, when the water level is very high, the presence of migratory species, dispersing over large distances, may homogenize fish communities. Such non-linear relationships have already been highlighted in birds where the community specialization index (a measure of the functional homogenization of communities) is maximal at intermediate values of fragmentation [34].

Regarding temporal variation in the number of species that contribute to beta diversity above the mean of the entire pool of species (i.e. SCBD values) we found a high contribution of species richness whereas species abundances and water level only had a marginal effect. More specifically, we found a positive relationship with species richness indicating that as species richness is increasing, communities within the lake tend to become more dissimilar. This can be explained by the presence of particular species with strong ecological requirements and/or poor dispersal abilities, confined to particular area of the lake. However, the relationship highlighted was non-linear and actually peaked for intermediate values of species richness. Thus, at very high richness communities tend to be more similar, which can be explained the widespread occurrence of species with low ecological requirements and/or strong dispersal abilities homogenizing communities at large spatial scale.

## Conclusion

The TSL is the largest inland fisheries in South-east Asia and supports the livelihood of 2.5 million people around the lake [8]. Its flood-pulse dynamic combined to the flow reversal of the TSR make it a unique system worldwide supporting high biodiversity by providing a large diversity of food and habitats for many birds and fishes. However, the growing demand for water for agricultural purposes and the construction of hydro-power dams along the Mekong river [8] combined to the effect of climate change is strongly threatening this system by altering and reducing flood intensity from 7% to 16% during the rainy season [35]. Such changes in the water regime are likely to have strong impacts on fish community composition by modifying several phenological events [36] such as the timing of migration or spawning and also by reducing the amount of submerged habitats upon which fish depends for growing and spawning. This may ultimately lead to a decrease in fish productivity and biodiversity. For instance, in 2016, hundred tons of brood-stock fish died within the conservation zone of Boeung Chhmar (which is temporarily connected to the lake) due to a prolonged drought. The strong spatio-temporal variations highlighted regarding the uniqueness of fish communities are likely to be the result of both spatial variation in environmental conditions and the seasonal migration of particular species which occurrence depends on the connectivity to floodplain habitats critical for their reproduction and survival. Promoting the connectivity to floodplain habitats is therefore an important step toward the maintenance of fish biodiversity and productivity upon which millions of people depend for their livelihood.

## Supporting information

S1 Table List of the 242 fish species captured among the six sampling sites during 141 weeks (from January 2012 to May 2014) spanning two and half hydrological cycles. (DOCX)

S2 Table List and characteristics of the 26 fish species that contributed to total beta diversity above the mean of the entire pool of species (according to [19,37]). The first column indicates species habitat requirements: (a) low wetland, (b) shallow sluggish or flowing and standing-water with aquatic vegetation, (c) floodplain throughout the middle and lower Mekong, (d) large and medium rivers and stream in the Mekong and Chao Phrya basins, (e) canals, ditches and reservoirs, (f) marine, freshwater, brackish and pelagic-neritic. The second column indicates species diet**:** (1) zooplankton, (2) crustaceans and mollusks, (3) insect, (4) algae and periphyton, (5) fish, (6) rotifers, (7) aquatic plants and fruits, (8) worm, (9) frogs (10) snakes. The third column indicates the species migratory strategy: Non migratory species (NM) and migratory species (M).
(DOCX)
