# Supplementary material for: Spatio-temporal variation of fish taxonomic composition in a South-East Asian flood-pulse system
Source: PLoS One. 2017 Mar 28;12(3):e0174582. doi: 10.1371/journal.pone.0174582 (PMC5370120; doi:10.1371/journal.pone.0174582)
Supplement: S1 Table — (DOCX) [file pone.0174582.s001.docx]

| Table S1. List of the 242 fish species captured among the six sampling sites during 141 weeks (from January 2012 to May 2014) spanning two and half hydrological cycles. | | | |
| --- | --- | --- | --- |
| Scientific name | Species abbreviation name | Genera name | Family name |
| *Aaptosyax grypus* | Aagr | Aaptosyax | Cyprinidae |
| *Acanthopsis spp.* | Acsp | Acanthopsoides | Cobitidae |
| *Acanthopsoides delphax* | Acde | Acanthopsoides | Cobitidae |
| *Acanthopsoides gracilentus* | *Acgr* | Acanthopsoides | Cobitidae |
| *Achiroides leucorhynchos* | Acle | Achiroides | Soleidae |
| *Achiroides melanorhynchus* | Acme | Achiroides | Soleidae |
| *Albulichthys albuloides* | Alal | Albulichthys | Cyprinidae |
| *Ambastaia sidthimunki* | Amsi | Ambastaia | Cobitidae |
| *Amblyrhynchichthys truncatus* | Amtr | Amblyrhynchichthys | Cyprinidae |
| *Anabas testudineus* | Ante | Anabas | Anabantidae |
| *Anguilla marmorata* | Anma | Anguilla | Anguillidae |
| *Apocryptodon madurensis* | Apma | Apocryptodon | Gobiidae |
| *Arius maculatus* | Arma | Arius | Ariidae |
| *Arius venosus* | Arve | Arius | Ariidae |
| *Bagarius bagarius* | Baba | Bagarius | Sisoridae |
| *Bagarius suchus* | Basu | Bagarius | Sisoridae |
| *Bagarius yarrelli* | Baya | Bagarius | Sisorinae |
| *Bagrichthys majusculus* | Bama | Bagrichthys | Bagridae |
| *Bagrichthys obscurus* | Baob | Bagrichthys | Bagridae |
| *Balitoropsis zollingeri* | Bazo | Balitoropsis | Balitoridae |
| *Bangana sp.* | Basp | Bangana | Cyprinidae |
| *Bangana yunnanensis* | Bayu | Bangana | Cyprinidae |
| *Barbichthys laevis* | Bala | Barbichthys | laevis |
| *Barbodes binotatus* | Babi | Barbodes | Cyprinidae |
| *Barbodes rhombeus* | Barh | Barbodes | Cyprinidae |
| *Barbonymus altus* | Baal | Barbonymus | Cyprinidae |
| *Barbonymus gonionotus* | Bago | Barbonymus | Cyprinidae |
| *Barbonymus schwanenfeldii* | Basc | Belodontichthys | Siluridae |
| *Belodontichthys truncatus* | Betr | Belodontichthys | Siluridae |
| *Boesemania microlepis* | Bomi | Boesemania | Sciaenidae |
| *Brachirus harmandi* | Brha | Brachirus | Soleidae |
| *Brachirus orientalis* | Bror | Brachirus | Soleidae |
| *Butis amboinensis* | Buam | Butis | Eleotridae |
| *Carcharhinus dussumieri* | Cadu | Carcharhinus | Carcharhinidae |
| *Catlocarpio siamensis* | Casi | Catlocarpio | Cyprinidae |
| *Channa gachua* | Chga | Channa | Channidae |
| *Channa lucius* | Chlu | Channa | Channidae |
| *Channa marulioides* | Chma | Channa | Channidae |
| *Channa marulius* | Chma.us | Channa | Channidae |
| *Channa micropeltes* | Chmi | Channa | Channidae |
| *Channa striata* | Chst | Channa | Channidae |
| *Chitala blanci* | Chbl | Channa | Channidae |
| *Chitala lopis* | Chlo | Chitala | [Notopteridae](http://www.aquaportail.com/taxonomie-famille-394-notopteridae.html) |
| *Chitala ornata* | Chor | Chitala | [Notopteridae](http://www.aquaportail.com/taxonomie-famille-394-notopteridae.html) |
| *Cirrhinus cirrhosus* | Cici | Cirrhinus | [Cyprinidae](http://www.fishbase.se/summary/FamilySummary.php?ID=122) |
| *Cirrhinus jullieni* | Ciju | Cirrhinus | [Cyprinidae](http://www.fishbase.se/summary/FamilySummary.php?ID=122) |
| *Cirrhinus microlepis* | Cimi | Cirrhinus | [Cyprinidae](http://www.fishbase.se/summary/FamilySummary.php?ID=122) |
| *Cirrhinus molitorella* | Cimo | Cirrhinus | [Cyprinidae](http://www.fishbase.se/summary/FamilySummary.php?ID=122) |
| *Clarias batrachus* | Clba | Clarias | Clariidae |
| *Clarias cataractus* | Clca | Clarias | Clariidae |
| *Clarias gariepinus* | Clga | Clarias | Clariidae |
| *Clarias macrocephalus* | Clma | Clarias | Clariidae |
| *Clarias meladerma* | Clme | Clarias | Clariidae |
| *Clarias sp.* | Clsp | Clarias | Clariidae |
| *Clupisoma longianalis* | Cllo | Clupisoma | Ailiidae |
| *Coilia lindmani* | Coli | Coilia | Engraulidae |
| *Coilia macrognathos* | Coma | Coilia | Engraulidae |
| *Corica soborna* | Coso | Corica | Clupeidae |
| *Cosmochilus harmandi* | Coha | Cosmochilus | Cyprinidae |
| *Crossocheilus atrilimes* | Crat | Crossocheilus | Cyprinidae |
| *Cyclocheilichthys apogon* | Cyap | Cyclocheilichthys | Cyprinidae |
| *Cyclocheilichthys armatus* | Cyar | Cyclocheilichthys | Cyprinidae |
| *Cyclocheilichthys enoplos* | Cyen | Cyclocheilichthys | Cyprinidae |
| *Cyclocheilichthys heteronema* | Cyhe | Cyclocheilichthys | Cyprinidae |
| *Cyclocheilichthys lagleri* | Cyla | Cyclocheilichthys | Cyprinidae |
| *Cyclocheilichthys repasson* | Cyre | Cyclocheilichthys | Cyprinidae |
| *Cyclocheilos furcatus* | Cyfu | Cyclocheilichthys | Cyprinidae |
| *Cynoglossus feldmanni* | Cyfe | Cynoglossus | Cynoglossidae |
| *Cynoglossus microlepis* | Cymi | Cynoglossus | Cynoglossidae |
| *Cyprinus carpio* | Cyca | Cyprinus | Cyprinidae |
| *Datnioides polata* | Dapo | Datnioides | Labotidae |
| *Datnioides undecimradiatus* | Daun | Datnioides | Labotidae |
| *Devario leptos* | Dele | Devario | Cyprinidae |
| *Discherodontus ashmeadi* | Dias | Discherodontus | Cyprinidae |
| *Discherodontus parvus* | Dipa | Discherodontus | Cyprinidae |
| *Esomus metallicus* | Esme | Esomus | Cyprinidae |
| *Folifer brevifilis* | Fobr | Folifer | Cyprinidae |
| *Gambusia affinis* | Gaaf | Gambusia | Poeciliidae |
| *Garra fasciacauda* | Gafa | Garra | Cyprinidae |
| *Glossogobius aureus* | Glau | Glossogobius | Cobitidae |
| *Glossogobius giuris* | Glgi | Glossogobius | Cobitidae |
| *Glyptothorax fuscus* | Glfu | Glyptothorax | Sisoridae |
| *Glyptothorax horai* | Glho | Glyptothorax | Sisoridae |
| *Glyptothorax laosensis* | Glla | Glyptothorax | Sisoridae |
| *Gymnothorax tile* | Gyti | Gymnothorax | Muraenidae |
| *Gyrinocheilus pennocki* | Gype | Gyrinocheilus | Gyrinocheilidae |
| *Hampala dispar* | Hadi | Hampala | Cyprinidae |
| *Hampala macrolepidota* | Hama | Hampala | Cyprinidae |
| *Helicophagus waandersii* | Hewa | Helicophagus | Pangasiidae |
| *Helostoma temminckii* | Hete | Helostoma | Helostomatidae |
| *Hemiarius stormii* | Hest | Hemiarius | Ariidae |
| *Hemibagrus filamentus* | Hefi | Hemibagrus | Bagridae |
| *Hemibagrus nemurus* | Hene | *Hemibagrus* | Bagridae |
| *Hemibagrus spilopterus* | Hesp | Hemibagrus | Bagridae |
| *Hemibagrus wyckii* | Hewy | Hemibagrus | Bagridae |
| *Hemibagrus wyckioides* | Hewy.des | Hemibagrus | Bagridae |
| *Hemimyzon pengi* | Hepe | Hemimyzon | Balitoridae |
| *Hemisilurus mekongensis* | Heme | Hemisilurus | Siluridae |
| *Henicorhynchus lobatus* | Helo | Henicorhynchus | Cyprinidae |
| *Henicorhynchus siamensis* | Hesi | Henicorhynchus | Cyprinidae |
| *Heteropneustes kemratensis* | Heke | Heteropneustes | Heteropneustidae |
| *Himantura undulata* | Hiun | Himantura | Dasyatidae |
| *Hypophthalmichthys molitrix* | Hymo | Hypophthalmichthys | Cyprinidae |
| *Hypophthalmichthys nobilis* | Hyno | Hypophthalmichthys | Cyprinidae |
| *Hyporhamphus limbatus* | Hyli | Hyporhamphus | Hemiramphidae |
| *Hypsibarbus lagleri* | Hyla | Hypsibarbus | Cyprinidae |
| *Hypsibarbus malcolmi* | Hyma | Hypsibarbus | Cyprinidae |
| *Hypsibarbus pierrei* | Hypi | Hypsibarbus | Cyprinidae |
| *Hypsibarbus suvattii* | Hysu | Hypsibarbus | Cyprinidae |
| *Hypsibarbus vernayi* | Hyve | Hypsibarbus | Cyprinidae |
| *Hypsibarbus wetmorei* | Hywe | Hypsibarbus | Cyprinidae |
| *Incisilabeo behri* | Inbe | Incisilabeo | Cyprinidae |
| *Kryptopterus cryptopterus* | Krcr | Kryptopterus | Siluridae |
| *Kryptopterus schilbeides* | Krsc | Kryptopterus | Siluridae |
| *Labeo chrysophekadion* | Lach | Labeo | Cyprinidae |
| *Labeo dyocheilus* | Lady | Labeo | Cyprinidae |
| *Labeo rohita* | Laro | Labeo | Cyprinidae |
| *Labiobarbus lineatus* | Lali | Labiobarbus | Cyprinidae |
| *Labiobarbus siamensis* | Lasi | Labiobarbus | Cyprinidae |
| *Labiobarbus sp. cf. lineatus* | Lasp | Labiobarbus | Cyprinidae |
| *Laides longibarbis* | Lalo | Laides | Schilbeidae |
| *Laubuka laubuca* | Lala | [Laubuka](https://en.wikipedia.org/wiki/Laubuka) | Cyprinidae |
| *Leptobarbus hoevenii* | Leho | Leptobarbus | Cyprinidae |
| *Lobocheilos melanotaenia* | Lome | Lobocheilos | Cyprinidae |
| *Lobocheilos rhabdoura* | Lorh | Lobocheilos | Cyprinidae |
| *Longiculter siahi* | Losi | Longiculter | Cyprinidae |
| *Luciosoma bleekeri* | Lubl | Luciosoma | Cyprinidae |
| *Lycothrissa crocodilus* | Lycr | Lycothrissa | Engraulidae |
| *Macrochirichthys macrochirus* | Mama | Macrochirichthys | Cyprinidae |
| *Macrognathus circumcinctus* | Maci | Macrognathus | Mastacembelidae |
| *Macrognathus siamensis* | Masi | Macrognathus | Mastacembelidae |
| *Mastacembelus armatus* | Maar | Mastacembelus | Mastacembelidae |
| *Megalops cyprinoides* | Mecy | Megalops | Magalopida |
| *Mekongina erythrospila* | Meer | Mekongina |  |
| *Micronema cheveyi* | Mich | Micronema | Siluridae |
| *Micronema hexapterus* | Mihe | Micronema | Siluridae |
| *Misgurnus anguillicaudatus* | Mian | Micronema | Siluridae |
| *Monopterus albus* | Moal | Monopterus | Synbranchidae |
| *Mystacoleucus obtusirostris* | Myob | Mystacoleucus | Cyprinidae |
| *Mystus albolineatus* | Myal | Mystus | Bagridae |
| *Mystus atrifasciatus* | Myat | Mystus | Bagridae |
| *Mystus bocourti* | Mybo | Mystus | Bagridae |
| *Mystus mysticetus* | Mymy | Mystus | Bagridae |
| *Mystus singaringan* | Mysi | Mystus | Bagridae |
| *Mystus wolffii* | Mywo | Mystus | Bagridae |
| *Nemapteryx nenga* | Nene | Nemapteryx | Ariidae |
| *Neolissochilus blanci* | Nebl | Neolissochilus | Cyprinidae |
| *Netuma thalassina* | Neth | netuma | Ariidae |
| *Notopterus notopterus* | Nono | Notopterus | Notopteridae |
| *Ompok bimaculatus* | Ombi | Ompok | Siluridae |
| *Ompok hypophthalmus* | Omhy | Ompok | Siluridae |
| *Ophisternon bengalense* | Opbe | Ophisternon | Synbranchidae |
| *Osphronemus exodon* | Osex | Osphronemus | Osphronemidae |
| *Osphronemus goramy* | Osgo | Osphronemus | Osphronemidae |
| *Osteochilus lini* | Osli | Osteochilus | Cyprinidae |
| *Osteochilus melanopleurus* | Osme | Osteochilus | Cyprinidae |
| *Osteochilus microcephalus* | Osmi | Osteochilus | Cyprinidae |
| *Osteochilus schlegeli* | Ossc | Osteochilus | Cyprinidae |
| *Osteochilus vittatus* | Osvi | Osteochilus | Cyprinidae |
| *Osteochilus waandersii* | Oswa | Osteochilus | Cyprinidae |
| *Osteogeneiosus militaris* | Osmi.ris | Osteogeneiosus | Ariidae |
| *Oxyeleotris marmorata* | Oxma | Oxyeleotris | Eleotridae |
| *Pangasianodon gigas* | Pagi | Pangasianodon | [Pangasiidae](https://fr.wikipedia.org/wiki/Pangasiidae) |
| *Pangasianodon hypophthalmus* | Pahy | Pangasianodon | [Pangasiidae](https://fr.wikipedia.org/wiki/Pangasiidae) |
| *Pangasius bocourti* | Pabo | Pangasius | [Pangasiidae](https://fr.wikipedia.org/wiki/Pangasiidae) |
| *Pangasius conchophilus* | Paco | Pangasius | [Pangasiidae](https://fr.wikipedia.org/wiki/Pangasiidae) |
| *Pangasius djambal* | Padj | Pangasius | [Pangasiidae](https://fr.wikipedia.org/wiki/Pangasiidae) |
| *Pangasius krempfi* | Pakr | Pangasius | [Pangasiidae](https://fr.wikipedia.org/wiki/Pangasiidae) |
| *Pangasius kunyit* | Paku | Pangasius | [Pangasiidae](https://fr.wikipedia.org/wiki/Pangasiidae) |
| *Pangasius larnaudii* | Pala | Pangasius | [Pangasiidae](https://fr.wikipedia.org/wiki/Pangasiidae) |
| *Pangasius macronema* | Pama | Pangasius | [Pangasiidae](https://fr.wikipedia.org/wiki/Pangasiidae) |
| *Pangasius nasutus* | Pana | Pangasius | [Pangasiidae](https://fr.wikipedia.org/wiki/Pangasiidae) |
| *Pangasius polyuranodon* | Papo | Pangasius | [Pangasiidae](https://fr.wikipedia.org/wiki/Pangasiidae) |
| *Pangasius spp.* | Pasp | Pangasius | [Pangasiidae](https://fr.wikipedia.org/wiki/Pangasiidae) |
| *Pao cambodgiensis* | Paca | Pao | Tetraodontidae |
| *Pao leiurus* | Pale | Pao | Tetraodontidae |
| *Parachela maculicauda* | Pama.cul | Parachela | Cyprinidae |
| *Parachela siamensis* | Pasi | Parachela | Cyprinidae |
| *Paralaubuca barroni* | Paba | Paralaubuca | Cyprinidae |
| *Paralaubuca riveroi* | Pari | Paralaubuca | Cyprinidae |
| *Paralaubuca typus* | Paty | Paralaubuca | Cyprinidae |
| *Parambassis apogonoides* | Paap | Parambassis | Ambassidae |
| *Parambassis siamensis* | Pasi.sis | Parambassis | Ambassidae |
| *Parambassis wolffii* | Pawo | Parambassis | Ambassidae |
| *Periophthalmodon septemradiatus* | Pese | Periophthalmodon | Gobiidae |
| *Phalacronotus apogon* | Phap | Phalacronotus | Siluridae |
| *Phalacronotus bleekeri* | Phbl | Phalacronotus | Siluridae |
| *Phalacronotus micronemus* | Phmi | Phalacronotus | Siluridae |
| *Piaractus brachypomus* | Pibr | Piaractus | [Serrasalmidae](https://fr.wikipedia.org/wiki/Serrasalmidae) |
| *Polynemus dubius* | Podu | Polynemus | Polynemidae |
| *Polynemus melanochir* | Pome | Polynemus | Polynemidae |
| *Polynemus multifilis* | Pomu | Polynemus | Polynemidae |
| *Poropuntius deauratus* | Pode | Poropuntius | Cyprinidae |
| *Pristis microdon* | Prmi | Pristis | Pristidae |
| *Pristolepis fasciata* | Prfa | Pristolepis | Pristolepididae |
| *Probarbus jullieni* | Prju | Pristolepis | Nandidae |
| *Probarbus labeamajor* | Prla | Probarbus | Cyprinidae |
| *Pseudolais micronemus* | Psmi | Pseudolais | Pangasiidae |
| *Pseudolais pleurotaenia* | Pspl | Pseudolais | Pangasiidae |
| *Pseudomystus siamensis* | Pssi | Pseudomystus | Bagridae |
| *Puntioplites bulu* | Pubu | Puntioplites | Cyprinidae |
| *Puntioplites falcifer* | Pufa | Puntioplites | Cyprinidae |
| *Puntioplites proctozysron* | Pupr | Puntioplites | Cyprinidae |
| *Puntioplites waandersi* | Puwa | Puntioplites | Cyprinidae |
| *Puntius brevis* | Pubr | Puntioplites | Cyprinidae |
| *Raiamas guttatus* | Ragu | Raiamas | Cyprinidae |
| *Rasbora borapetensis* | Rabo | Rasbora | Cyprinidae |
| *Rasbora myersi* | Ramy | Rasbora | Cyprinidae |
| *Rasbora paviana* | Rapa | Rasbora | Cyprinidae |
| *Rasbora tornieri* | Rato | Rasbora | Cyprinidae |
| *Rasbora trilineata* | Ratr | Rasbora | Cyprinidae |
| *Rasbosoma spilocerca* | Rasp | Rasbosoma | Cyprinidae |
| *Rhinogobius taenigena* | Rhta | Rhinogobius | Gobiidae |
| *Scaphognathops bandanensis* | Scba | Scaphognathops | Cyprinidae |
| *Scaphognathops stejnegeri* | Scst | Scaphognathops | Cyprinidae |
| *Schistura aramis* | Scar | Schistura | Namacheilidae |
| *Schistura athos* | Scat | Schistura | Namacheilidae |
| *Schistura crabro* | Sccr | Schistura | Namacheilidae |
| *Schistura daubentoni* | Scda | Schistura | Namacheilidae |
| *Schistura latifasciata* | Scla | Schistura | Namacheilidae |
| *Scleropages formosus* | Scfo | Scleropages | Osteoglossidae |
| *Sikukia gudgeri* | Sigu | Sikukia | Cyprinidae |
| *Syncrossus beauforti* | Sybe | Syncrossus | Cobitidae |
| *Syncrossus helodes* | Syhe | Syncrossus | Cobitidae |
| *Systomus rubripinnis* | Syru | Tenualosa | Clupeidae |
| *Tenualosa thibaudeaui* | Teth | Tenualosa | Clupeidae |
| *Tenualosa toli* | Teto | Tenualosa | Clupeidae |
| *Thynnichthys thynnoides* | Thth | Thynnichthys | Cyprinidae |
| *Tor laterivittatus* | Tola | Tor | Cyprinidae |
| *Tor sinensis* | Tosi | Tor | Cyprinidae |
| *Tor tambroides* | Tota | Tor | Cyprinidae |
| *Toxotes chatareus* | Toch | Toxotes | Toxotidae |
| *Toxotes microlepis* | Tomi | Toxotes | Toxotidae |
| *Trichopodus microlepis* | Trmi | Trichogaster | [Osphronemidae](http://www.aquaportail.com/taxonomie-famille-123-osphronemidae.html) |
| *Trichopodus pectoralis* | Trpe | Trichogaster | [Osphronemidae](http://www.aquaportail.com/taxonomie-famille-123-osphronemidae.html) |
| *Trichopodus trichopterus* | Trtr | Trichogaster | [Osphronemidae](http://www.aquaportail.com/taxonomie-famille-123-osphronemidae.html) |
| *Wallago attu* | Waat | Wallago | Siluridae |
| *Wallago leerii* | Wale | Wallago | Siluridae |
| *Xenentodon cancila* | Xeca | Xenentodon | Belonidae |
| *Yasuhikotakia eos* | Yaeo | Yasuhikotakia | Botiidae |
| *Yasuhikotakia lecontei* | Yale | Yasuhikotakia | Botiidae |
| *Yasuhikotakia modesta* | Yamo | Yasuhikotakia | Botiidae |
