# Supplementary material for: Spatio-temporal variation of fish taxonomic composition in a South-East Asian flood-pulse system
Source: PLoS One. 2017 Mar 28;12(3):e0174582. doi: 10.1371/journal.pone.0174582 (PMC5370120; doi:10.1371/journal.pone.0174582)
Supplement: S2 Table — The first column indicates species habitat requirements: (a) low wetland, (b) shallow sluggish or flowing and standing-water with aquatic vegetation, (c) floodplain throughout the middle and lower Mekong, (d) large and medium rivers and stream in the Mekong and Chao Phrya basins, (e) canals, ditches and reservoirs, (f) marine, freshwater, brackish and pelagic-neritic. The second column indicates species diet: (1) zooplankton, (2) crustaceans and mollusks, (3) insect, (4) algae and periphyton, (5) fish, (6) rotifers, (7) aquatic plants and fruits, (8) worm, (9) frogs (10) snakes. The third column indicates the species migratory strategy: Non migratory species (NM) and migratory species (M). (DOCX) [file pone.0174582.s002.docx]

| **Table S2.** List and characteristics of the 26 fish species that contributed to total beta diversity above the mean of the entire pool of species (according to [32,33]). The first column indicates species habitat requirements: (a) low wetland, (b) shallow sluggish or flowing and standing-water with aquatic vegetation, (c) floodplain throughout the middle and lower Mekong, (d) large and medium rivers and stream in the Mekong and Chao Phrya basins, (e) canals, ditches and reservoirs, (f) marine, freshwater, brackish and pelagic-neritic. The second column indicates species feeding behavior**:** (1) zooplankton, (2) crustaceans and mollusks, (3) insect, (4) algae and periphyton, (5) fish, (6) rotifers, (7) aquatic plants and fruits, (8) worm, (9) frogs (10) snakes. The third column indicates the species migratory strategy: Non migratory species (NM) and migratory species (M). | | | |
| --- | --- | --- | --- |
| Species | Habitat | Feeding behavior | Migration pattern |
| *Parambassis wolffi* | b, c | 2, 3, 5 | NM |
| *Henicorhynchus siamensis* | d | 4 | M |
| *Labiobarbus lineatus* | d | 1, 4 | M |
| *Mystus bocourti* | d | 2 | NM |
| *Mystus albolineatus* | b | 1, 3, 5 | NM |
| *Henicorhynchus lobatus* | d | 4 | M |
| *Cyclocheilichthys armatus* | d | 1, 2, 3 | NM |
| *Cyclocheilichthys enoplos* | c, d | 2, 3, 4, 5, 7 | M |
| *Mystus singaringan* | b | 1, 3, 5 | NM |
| *Pristolepis fasciata* | b | 2, 3, 4, 7 | NM |
| *Trichopodus microlepis* | c | 1, 2, 3 | NM |
| *Labeo chrysophekadion* | b, c | 4,7 | M |
| *Osteochilus vittatus* | d, e | 4 | NM |
| *Paralaubuca typus* | d | 1, 3 | M |
| *Hypsibarbus lagleri* | d, c | 1, 4, 8 | M |
| *Xenentodon cancila* | f | 3, 5 | M |
| *Parachela maculicauda* | c, d | 1, 3 | NM |
| *Puntioplites falcifer* | d | 3, 7 | NM |
| *Labiobarbus siamensis* | d | 1, 4 | M |
| *Anabas testudineus* | e, c | 2, 4,5 | NM |
| *Notopterus notopterus* | b, e, f | 2, 3, 5, 7 | NM |
| *Thynnichthys thynnoides* | c, d | 1, 4 | M |
| *Rasbora tornieri* | e | 3 | NM |
| *Channa striata* | a, b, c, e | 2, 3, 4, 8, 9,10 | NM |
